# Sparse Regression Based Structure Learning of Stochastic Reaction Networks from Single Cell Snapshot Time Series

**DOI:** 10.1371/journal.pcbi.1005234

**Published:** 2016-12-06

**Authors:** Anna Klimovskaia, Stefan Ganscha, Manfred Claassen

**Affiliations:** 1 Institute for Molecular Systems Biology, ETH Zurich, Zurich, Switzerland; 2 Swiss Institute of Bioinformatics, Zurich, Switzerland; 3 Life Science Zurich Graduate School, Zurich, Switzerland; University of Michigan, UNITED STATES

## Abstract

Stochastic chemical reaction networks constitute a model class to quantitatively describe dynamics and cell-to-cell variability in biological systems. The topology of these networks typically is only partially characterized due to experimental limitations. Current approaches for refining network topology are based on the explicit enumeration of alternative topologies and are therefore restricted to small problem instances with almost complete knowledge. We propose the *reactionet lasso*, a computational procedure that derives a stepwise sparse regression approach on the basis of the Chemical Master Equation, enabling large-scale structure learning for reaction networks by implicitly accounting for billions of topology variants. We have assessed the structure learning capabilities of the reactionet lasso on synthetic data for the complete TRAIL induced apoptosis signaling cascade comprising 70 reactions. We find that the reactionet lasso is able to efficiently recover the structure of these reaction systems, ab initio, with high sensitivity and specificity. With only < **1**% false discoveries, the reactionet lasso is able to recover 45% of all true reactions ab initio among > **6000** possible reactions and over **10**^**2000**^ network topologies. In conjunction with information rich single cell technologies such as single cell RNA sequencing or mass cytometry, the reactionet lasso will enable large-scale structure learning, particularly in areas with partial network structure knowledge, such as cancer biology, and thereby enable the detection of pathological alterations of reaction networks. We provide software to allow for wide applicability of the reactionet lasso.

## Author Summary

Virtually all biological processes are driven by biochemical reactions. However, their quantitative description in terms of stochastic chemical reaction networks is often precluded by the computational difficulty of structure learning, i.e. the identification of biologically active reaction networks among the combinatorially many possible topologies. This work describes the *reactionet lasso*, a structure learning approach that takes advantage of novel, information-rich single cell data and a tractable problem formulation to achieve structure learning for problem instances hundreds of orders of magnitude larger than previously reported. This approach opens the prospect of obtaining quantitative and predictive reaction models in many areas of biology and medicine, and in particular areas such as cancer biology, which are characterized by significant system alterations and many unknown reactions.

This is a *PLOS Computational Biology* Methods paper.

## Introduction

Cellular processes are essentially implemented by networks of biochemical reactions. The topology of such networks is typically only partially known, rendering the identification of the correct network from experimental data a key challenge. Despite the importance of this task, only little progress has been made in devising methods to systematically and comprehensively infer topologies of non-trivial chemical reaction networks. In this work, we propose a sparse regression approach tailored to the task of large-scale model selection for chemical reaction networks.

Different model classes have been developed to describe biochemical reaction systems. In order of increasing level of detail these comprise statistical time series models, such as autoregressive models and dynamic Bayesian networks, deterministic ordinary differential equation or stochastic differential equation based kinetic models [1]. The choice of model class depends on prior information for the system of interest and type of experimental data. Single cell technologies furnish further statistical information about component distributions, e.g. variances and covariances, aiding in systems identification [2] and are expected to become increasingly prevalent in routine biological research [3].

Two main computational tasks arise when learning any of these models from data: parameter inference, and structure learning. Parameter inference aims at finding model parameters (e.g. kinetic rate constants). Parameter inference has been performed by sampling from posterior parameter distributions, or global non-convex or convex optimization methods [4]. Structure learning aims at additionally identifying the reaction network topology governing the dynamics of the system components.

Parameter inference becomes increasingly computationally intensive for larger systems with numerous parameters [1]. Structure learning for these systems is an even more daunting task since parameter inference has to be performed for each of the possibly very many different system topologies. Therefore, structure learning is typically confined to comparison of a small, carefully selected set of candidate topologies by means of model selection criteria, such as information criteria (e.g. AIC, BIC) or Bayes Factors [5–7]. However, this approach requires substantial prior knowledge about the studied system in order to identify reasonable candidate models. Systematic approaches to enumerate a subset of sensible topologies have not been reported until recently. These approaches implement greedy strategies that either iteratively reduce the number of reactions of an overcomplete system of reactions or add reactions one at a time to a system with a minimal set of reactions [8]. However, such greedy approaches do not guarantee finding globally optimal topologies for non-convex fitting objectives. Furthermore, exploration of the multitude of local optima in the context of combinatorially many possible topologies becomes computationally prohibitive due to the requirement to explicitly evaluate every considered candidate topology. No global approaches have been reported to perform structure learning by comprehensively evaluating model candidates for stochastic chemical reaction networks.

We propose the *reactionet lasso*, a convex relaxation of the structure learning task. This approach yields a single best sparse reaction set from all possible reactions by translating a recent sparse identification approach for nonlinear dynamic systems [9] to operate on and deal with non-trivial application specific parameter and noise structure for time series snapshot data acquired for stochastic chemical reaction networks.

## Results

### Sparse regression for structure learning of stochastic reaction networks

Structure learning by the reactionet lasso takes advantage of the formal link between the chemical reaction model and the observed data that is defined by the Chemical Master Equation. This differential equation system describes the temporal evolution of the abundance distributions of species governed by a stochastic chemical reaction network [10]. The moment generating functions of the Chemical Master Equation give rise to the moment equations, a system of ordinary differential equations for the temporal evolution of the central moments *M*_**r**_ of the abundance distributions (see Methods).

$$\begin{array}{c} {\dot{M}}_{r}=\Sigma_{l}k_{l}F_{rl}\left(t;M\right), \end{array}$$ (1)

with rate constants *k*_*l*_, time *t* and set of all central moments of individual species **M**. For mass action kinetics the terms *F*_**r***l*_(*t*; **M**) are polynomials over these moments such as abundance means and variances of individual species. *F*_**r***l*_(*t*; **M**) will be referred to as stoichiometric moment functions herein (see also S1 Text).

The moment equations constitute the formal link between the time series snapshot data and the rate constants of the underlying chemical reactions. Rate constant estimation for stochastic mass action kinetics reaction networks in this context therefore reduces to parameter estimation for the ordinary differential equation system [Eq 1] with stoichiometric moment functions determined from the time series data.

Parameter estimation for a mass action kinetics network typically requires the costly integration of the moment equations for every considered parameter configuration. Imputation of the moment gradients by gradient matching procedures (see Methods) circumvents these type of evaluations and, in conjunction with the empirical moments, allows for parameter inference by means of a non-negative linear regression task with the least squares estimate $\hat{k}$ for rate constants **k** given by:

$$\begin{array}{c} \hat{k}=\arg\min_{k\geq 0}{∥\hat{b}-\hat{A}k∥}_{2}^{2}, \end{array}$$ (2)

where the response vector elements *b*_*j*_ corresponds to the vector of empirical gradient estimates for ${\dot{M}}_{j}\left(t\right)$ from the gradient matching procedure (see Methods) and the design matrix entries ${\hat{A}}_{jl}$ correspond to the estimates of the stoichiometric moment functions *F*_*jl*_(*t*; **M**):

$$b=\left[\begin{array}{c} {\hat{\dot{M}}}_{r_{1}}\left(t_{1}\right)\\ \vdots \\ {\hat{\dot{M}}}_{r_{1}}\left(t_{T}\right)\\ {\hat{\dot{M}}}_{r_{2}}\left(t_{1}\right)\\ \vdots \\ {\hat{\dot{M}}}_{r_{2}}\left(t_{T}\right)\\ \vdots \\ {\hat{\dot{M}}}_{r_{N}}\left(t_{1}\right)\\ \vdots \\ {\hat{\dot{M}}}_{r_{N}}\left(t_{T}\right) \end{array}\right],A=\left[\begin{array}{cccc} {\hat{F}}_{r_{1}1}\left(t_{1}\right) & {\hat{F}}_{r_{1}2}\left(t_{1}\right) & \cdots & {\hat{F}}_{r_{1}L}\left(t_{1}\right)\\ \vdots & \vdots & \vdots & \vdots \\ {\hat{F}}_{r_{1}1}\left(t_{T}\right) & {\hat{F}}_{r_{1}2}\left(t_{T}\right) & \cdots & {\hat{F}}_{r_{1}L}\left(t_{T}\right)\\ {\hat{F}}_{r_{2}1}\left(t_{1}\right) & {\hat{F}}_{r_{2}2}\left(t_{1}\right) & \cdots & {\hat{F}}_{r_{2}L}\left(t_{1}\right)\\ \vdots & \vdots & \vdots & \vdots \\ {\hat{F}}_{r_{2}1}\left(t_{T}\right) & {\hat{F}}_{r_{2}2}\left(t_{T}\right) & \cdots & {\hat{F}}_{r_{2}L}\left(t_{T}\right)\\ \vdots & \vdots & \vdots & \vdots \\ {\hat{F}}_{r_{N}1}\left(t_{1}\right) & {\hat{F}}_{r_{N}2}\left(t_{1}\right) & \cdots & {\hat{F}}_{r_{N}L}\left(t_{1}\right)\\ \vdots & \vdots & \vdots & \vdots \\ {\hat{F}}_{r_{N}1}\left(t_{T}\right) & {\hat{F}}_{r_{N}2}\left(t_{T}\right) & \cdots & {\hat{F}}_{r_{N}L}\left(t_{T}\right) \end{array}\right].$$

This linear regression formulation has been applied for parameter inference of deterministic chemical reaction models [6, 11].

Model selection across small sets of model variants has previously been performed with information criteria [11] or model averaging [6]. The Lasso constitutes another approach for efficient and comprehensive model selection in linear regression models [12]. It introduces an L1 norm (‖ ⋅ ‖_1_) regularization on the parameters **k** to promote the identification of sparse solutions, i.e. solutions with many zero-valued parameter estimates.

$$\begin{array}{c} \hat{k}=\arg\min_{k\geq 0}∥\hat{b}-\hat{A}{k∥}_{2}^{2}+\lambda {∥k∥}_{1}, \end{array}$$ (3)

Various extensions of the Lasso method were introduced in literature to improve its shrinkage properties in the presence or absence of heteroscedasticity [13].

While the Lasso has been used in recent reports to identify general nonlinear dynamical systems [9] or to select the mechanism types (mass action or Hill kinetics) of a fixed reaction set defined by the deterministic Repressilator comprising six components [14], it still remains to adapt the regression model and regularization concepts to enable more comprehensive model selection for realistic reaction systems that exhibit stochasticity and larger amount of components/reactions. The next sections will delineate in detail the challenges and solutions implemented in the reactionet lasso to achieve this goal.

### Reactionet lasso

**Fig 1:**
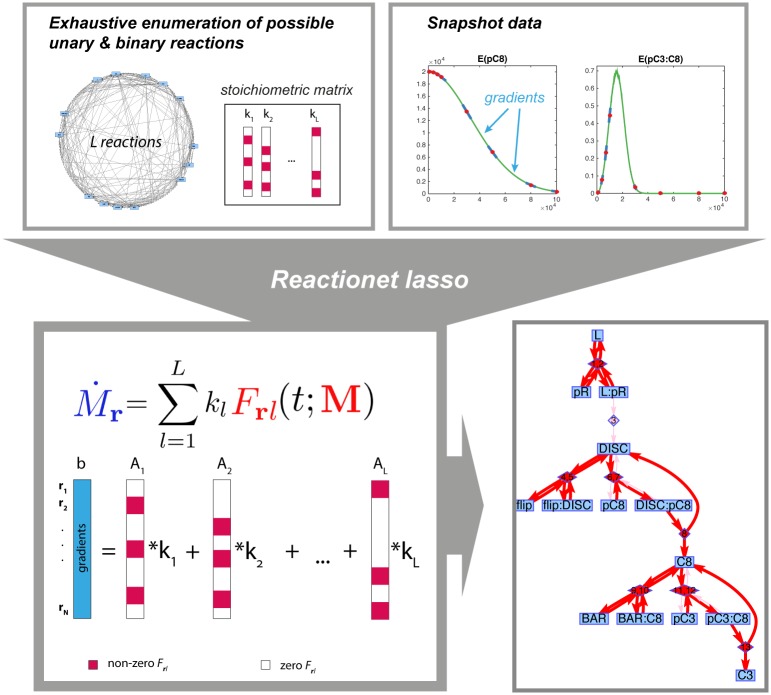
Schematic representation of *reactionet lasso* procedure. For details see section Methods.

This section introduces the reactionet lasso (Fig 1), a computational method for learning the structure of chemical reaction networks. The overarching strategy of this procedure consists of (1) enumerating all (or at least a significant fraction of reasonable) conceivable unary/binary reactions between the components of a reaction system of interest and (2) applying an appropriate stepwise sparse regression approach to select the sparse subset of reactions underlying the observed dynamics in the snapshot time series data.

The following properties of such structure learning instances preclude the application of conventional least squares based approaches for parameter estimation and selection: (1) noise and heteroscedasticity of the observed response $\hat{b}$ (empirical moment gradient estimates) as well as in the observed design matrix $\hat{A}$ (stoichiometric moment function evaluations) and (2) different scales of individual parameters *k*_*i*_ (rate constants) resulting from the occurrence of large a spectrum of fast and slow reactions. The reactionet lasso addresses each of these challenges in as delineated in the following.

The intrinsic variability of stochastic chemical kinetics result induces variability of the empirical estimates of moments and their gradients. Therefore the observed response vector as well as the stoichiometric moment functions in the design matrix are expected to deviate from the true latent correspondents. We capture this by defining $b=\hat{b}+\epsilon_{b}$ and $A=\hat{A}+\epsilon_{A}$ to be the true latent moment gradients and stoichiometric moment functions, and ***ϵ***_*A*_ and ***ϵ***_*b*_ to be their respective intrinsic variability induced deviations from the estimated/observed quantities. If we knew the true values of the latent variables, finding the rate constants **k** would translate to solving the following equation:

$$\begin{array}{c} b=Ak. \end{array}$$ (4)

By substituting the variables in eq 4 with the definitions for our empirical estimates of the latent variables we obtain:

$$\begin{array}{c} \hat{b}=\hat{A}k+\epsilon . \end{array}$$ (5)

with ***ϵ***: = ***ϵ***_*A*_ **k** − ***ϵ***_*b*_.

Eq 5 seems to motivate a straightforward optimization strategy to compute a maximum likelihood parameter estimate given the observations for moment gradients and stoichiometric moment functions (e.g. least squares for independent and normally distributed residuals ***ϵ***). However, it becomes apparent that this strategy is not valid due to the residual ***ϵ*** being a function of the parameters **k** (by virtue of the noise in the observed design matrix).

The reactionet lasso implements a stepwise strategy to address this dependency. The first step (Step 1) is a Feasible Generalized Least Squares (FG) estimate. It comprises the estimation of the variances of the residuals ***ϵ***_*b*_ and ***ϵ***_*A*_ via bootstrapping of the gradient estimates and stoichiometric moment functions on the basis the single-cell data. A preliminary least squares fit is then performed to achieve an estimate **k**^*LS*^ for eq 5. This estimate is expected to approximate the order of magnitude of the individual rate constants. In conjunction with the estimates of the variances of the residuals ***ϵ***_*b*_ and ***ϵ***_*A*_, we use **k**^*LS*^ to achieve an estimate of the component-wise variance $\Sigma_{\epsilon }=\text{diag}\left\{\sigma_{\epsilon_{1}}^{2},\ldots ,\sigma_{\epsilon_{R}}^{2}\right\}$ of the residuals ***ϵ***. To achieve this estimate we use only first order moments (means), as they are less subjected to noise in the design matrix and provide a more robust estimate of the covariance matrix *Σ*_*ϵ*_. This estimate will allow us to operate with the rescaled observed response vector ${\hat{b}}^{S}=\Sigma_{\epsilon }^{-1/2}\hat{b}$ and design matrix ${\hat{A}}^{S}=\Sigma_{\epsilon }^{-1/2}\hat{A}$ to adjust for heteroscedasticity and enable effective linear regression [15].

$$\begin{array}{c} {\hat{k}}^{FG}=\arg\min_{k\geq 0}{∥{\hat{b}}^{S}-{\hat{A}}^{S}k∥}_{2}^{2}, \end{array}$$ (6)

The subsequent steps aim at addressing the second challenge introduced above, i.e. the different scales of individual parameters *k*_*i*_, which render conventional sparse regression approaches (such as the Lasso) suboptimal due to the uniform penalization strength of the L1 norm ‖.‖_1_ across all components *k*_*i*_ of the parameter vector *k*. The adaptive Lasso [16] constitutes an alternative to the conventional Lasso. It defines a regularization penalty that is scaled component-wise by the expected order of magnitude $\hat{k_{i}}$ of the respective component *i*.

In Step 2 of the reactionet lasso, we apply a combination of the adaptive and relaxed Lasso, stability selection based prioritization of reactions and an additional stepwise backward regression to achieve the final set of reported reactions. We use the parameter estimates from Step 1 (obtained with Moore–Penrose pseudoinverse matrix), i.e. $\tilde{k}={\hat{k}}^{FG}$, in order to adapt the regularization penalty.

To improve shrinkage, the adaptive Lasso is followed by a relaxed Lasso [17] that recomputes optimal parameter estimates with respect to the objective specified in eq 6, while only considering the set $\Phi =\{l:k_{l}^{FG}\neq 0\}$ of parameters that were not set to zero in Step 1, for which the optimal solution is

$$\begin{array}{c} {\hat{k}}^{ARL}=\arg\min_{k\geq 0}∥{\hat{b}}^{S}-{\hat{A}}^{S,\Phi }{k∥}_{2}^{2}+\lambda \Sigma_{i}\left|k_{i}/{\tilde{k}}_{i}\right|, \end{array}$$ (7)

where ${\hat{A}}^{S,\Phi }$ contains only that columns of ${\hat{A}}^{S}$, which are in a set *Φ*.

The adaptive relaxed Lasso solution has been computed by optimizing the respective Alternating Direction Method of Multipliers (ADMM) formulations [18]. The adaptive relaxed Lasso is performed with five fold cross validation. We used stability selection to prioritize reactions according to their frequency of being selected across all cross validation folds [19]. Bayesian information criterion (BIC) was used as selection criterion (S2 Text).

In summary, the reactionet lasso procedure constitutes a stepwise sparse regression approach that addresses the parameter-dependent noise and heteroscedasticity in the response and design matrix for structure learning of stochastic chemical reaction systems. See also Fig 1 for a schematic overview of its steps. Software implementing the reactionet lasso can be found at http://www.imsb.ethz.ch/research/claassen/Software/reactionet_lasso.html.

### Ab initio structure learning of chemical reaction networks

We first consider an extreme and yet conceptually simple scenario where we aim at learning the structure of a reaction network without any prior knowledge about the underlying reactions. While this scenario rarely occurs in a real world application because typically some prior knowledge of relevant reactions is available, we first investigate this scenario to demonstrate the structure learning capabilities of the reactionet lasso.

We study two systems varying in number of components and reactions: (1) the enzymatic reaction system with four components and three reactions, (2) the receptor subunit of a recently reported kinetic model of TRAIL induced apoptosis with fourteen components and thirteen reactions, which can be combined in a total of 2275 possible unary or binary reactions, giving a total of more than 10^600^ possible reaction network candidates. For these systems we simulated 5 replicates each with either 10^3^, 10^4^ or 10^5^ single cell trajectories with the stochastic simulation algorithm [20]. We then generated snapshot time series datasets from the single cell trajectories by defining pools of cells at selected sets of 7, 13, or 28 time points. Moment gradients were estimated either with the smoothing procedure, cubic splines or the finite difference scheme (see Methods).

**Fig 2:**
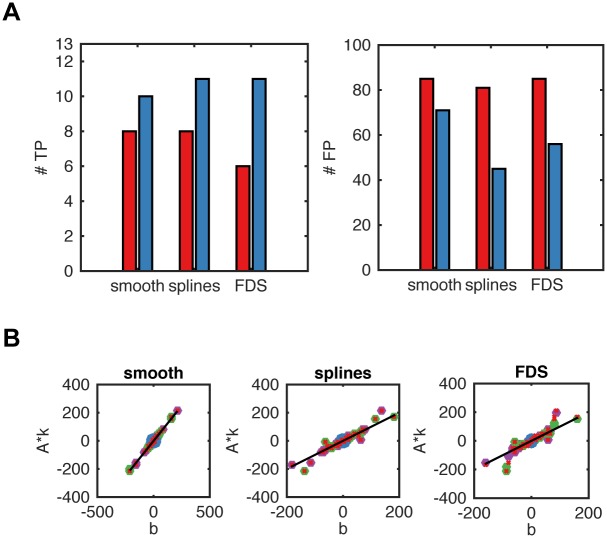
Performance assessment of the first *reactionet lasso* step for 10^5^ single cell trajectories of the apoptotic receptor subunit. (A) Enrichment and depletion of true and respectively false positive reactions for the reaction rate estimates ${\hat{k}}^{LS}$ (red) and ${\hat{k}}^{FG}$ (blue). Results are reported for gradient estimation procedures smooth, FDS, splines (see main text for details). (B) Comparison of response (empirical moment gradients) and prediction with feasible generalized least square estimate for moments of different order: means (blue), variances (green), covariances (yellow) and prediction with true rate constants for all moments (red crosses).

The reactionet lasso achieves structure learning of chemical reaction networks via a two step sparse regression formulation that (1) specifically accounts for heteroscedasticity in the response vector and the design matrix of the regression instances and (2) assumes a regularizer that encourages sparse reaction sets by suppressing compensatory reaction sets with small rate constants (Fig 1). The first step aims at accounting for heteroscedasticity and, most importantly at reducing the number of reaction candidates for the second step that both capture the empirical moment gradients and select for correct reactions (Fig 2). The following results are based on moment equations for all moments up to order two, i.e. means, variances and covariances. Following Step 1 of the reactionet lasso, we achieve a substantial reduction to less than 100 candidate reactions that, regardless of the moment gradient estimation technique, retains at least ten of the thirteen true reactions (Fig 1A). The vast majority of the empirical moment gradients are well fit by the set of candidate reactions. The few moment gradients that are suboptimally captured correspond to higher order moments such as variances or covariances whose highly dynamic behavior precluded accurate gradient estimation by either the finite difference or spline fit. (Fig 2B). Step 2 of the reactionet lasso procedure uses a relaxed adaptive Lasso estimator to estimate the rate constants of a sparse set of candidate reactions following from Step 1. The method recovers ten out of thirteen reactions correctly with one false positive reaction when assuming no prior knowledge and selecting a suitable model with BIC (S1A Fig). Similar performance is achieved for the enzymatic reaction network (S1B Fig). These results demonstrate that the stepwise sparse regression strategy of Step 2 completes the structure learning task from the candidate reactions supplied by Step 1 with great sensitivity and specificity. In summary, the reactionet lasso is able to ab initio reconstruct the reaction network structure of typically-sized signaling cascades such as the fourteen component receptor subunit of TRAIL induced apoptosis [21].

We further evaluated the impact of different gradient estimation approaches on structure learning performance (S2–S4 Figs). For benchmarking purposes we used the smoothed empirical moment gradient estimate as a ground truth which is not available in a real time series snapshot setting. According to these considerations, the cubic spline estimator achieves almost optimal performance for thirteen or more time points, whereas FDS is consistently inferior. These results indicate that the cubic spline estimator provides the most favorable structure learning performance for empirical moment gradients.

**Fig 3:**
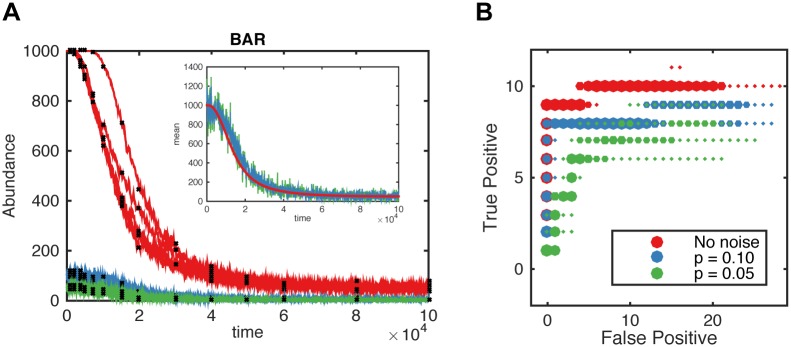
Influence of measurement noise on structure learning capacity. (A) Example of several single cell trajectories of one of the species (BAR) in apoptotic receptor subunit: without measurement noise (red), with measurement noise according to the binomial model with probability of success *p* = 0.1 (blue), 0.05 (green). Comparison of reconstructed means for known *p* between different noise levels shows how empirical moments are affected by measurement noise. Black dots represent snapshot measurements used for the inference procedure. (B) Overlay of five regularization paths in terms of true/false positive tradeoff for different measurement noise levels as indicated in the legend in terms of binomial capture efficiency. Structure learning performance for 10^5^ single cell trajectories and thirteen time points of the apoptotic receptor subunit. Empirical moment gradients estimated with splines.

We evaluated how measurement noise affects the ability of reactionet lasso to learn the reaction network structure. We assume a binomial measurement noise model that reflects the incomplete capture efficiency inherent to all single cell technologies (see Methods, S3 Text). While structure learning performance is reduced with increasing levels of measurement noise, the reactionet lasso still recovers more than 50% of the reactions for the apoptotic receptor subunit at levels reported for single cell sequencing and mass cytometry approaches (Fig 3, S5 Fig).

**Fig 4:**
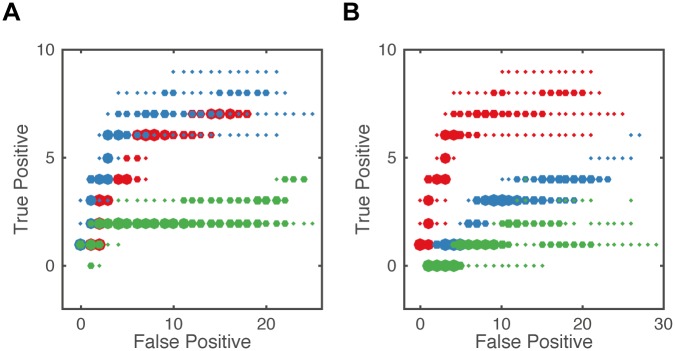
Overlay of five regularization paths in terms of true/false positive tradeoff over different data availability situations. Results for *reactionet lasso* application to apoptotic receptor subunit (*p* = 0.05). Empirical moment gradients estimated with cubic splines. (A) 10^5^ single cell trajectories evaluated at different amount of time points: 28 (red), 13 (blue), 7 (green). (B) Different number of single cell trajectories: 10^5^ (red), 10^4^ (blue), 10^3^ (green) evaluated at thirteen time points.

To assess the relative importance of the amount of available data, we varied the amount of time points and single cell recordings used at each time point. Interestingly, we found that the inclusion of additional measurement time points did not improve structure learning performance. However, the tradeoff between true and false positive reaction discoveries worsened considerably with fewer time points (Fig 4A). While we found that decreasing the amount of single cell measurements per time point did result in noticeable performance losses, this situation does not constitute a limitation for flow cytometry techniques, that are easily able to generate millions of single cell snapshots (Fig 4B). Cell count related performance losses can be associated with higher absolute variability and therefore reduced accuracy of empirical moment estimates (S6 Fig). We conclude that careful selection of amount of single cell measurements and number as well as position of time points (S7 Fig) translates to accurate interpolation and subsequent gradient fitting, thereby leading to good structure learning performance of the reactionet lasso.

**Fig 5:**
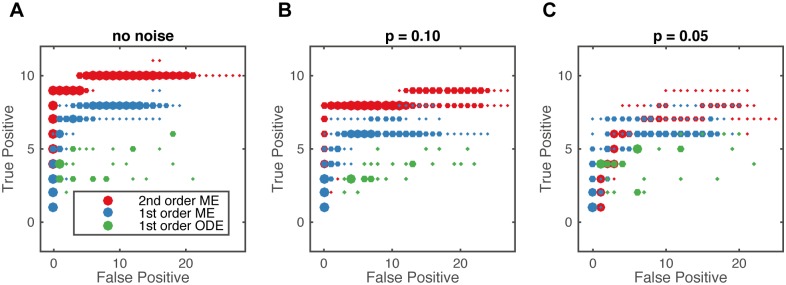
Overlay of five regularization paths in terms of true/false positive tradeoff for different moment orders of gradients considered for structure learning: up to 2nd order (red) Moment Equations, up to 1st order (blue) Moment Equations and only 1st order moments for deterministic mean ODE model (green). Structure learning performance for 10^5^ single cell trajectories and thirteen time points of the apoptotic receptor subunit. Empirical moment gradients estimated with splines. Results represented for different levels of measurement noise: (A) no noise; (B) *p* = 0.1; (C) *p* = 0.05.

We further investigated the impact of including different moment orders for structure learning. As expected, precisely estimated higher-order moments contain a substantial amount of information and therefore enhance the structure learning capability accordingly (Fig 5A). However, although this relationship still holds for medium levels of measurement noise (capture efficiency *p* = 0.1), (Fig 5B), the inclusion of second order moments becomes misleading for high levels of measurement noise (capture efficiency *p* = 0.05, Fig 5C). This observation is likely caused by the limited ability to accurately estimate higher order moments for high levels of measurement noise. However, the performance of the reactionet lasso assuming stochastic kinetics modeled with moment equations (higher order ME) is consistently better than assuming a deterministic kinetics modeled with mean based ordinary equations (1st order ODE). This observation demonstrates that the incorporation of higher order moment information induced by the chemical kinetics and accessible by means of single cell measurements allows for significantly improved structure learning capacity.

In summary, the benchmarks above strongly advocate for the use of an experimental setup that allows for sufficiently dense sampling across time to ensure accurate empirical moment gradient estimates, as well as single cell technology, such as flow/mass cytometry, which provide 10^4^ or more single cell measurements, for the accurate estimation of higher order moments. In these situations the reactionet lasso is capable of ab initio recovery of almost the complete reaction network structure with more than a dozen components.

### Structure learning of large chemical reaction networks with prior knowledge

We now consider a scenario where we aim at learning the structure of a large reaction network with partial knowledge about the underlying reactions. For this situation we demonstrate how reactionet lasso is capable of recovering a sizable amount of the unknown reactions, for a reaction network as large as the 70 reaction TRAIL induced apoptosis cascade [21].

Structure learning tasks for chemical reaction networks typically aim at complementing already available partial knowledge on reaction sets. We assessed the ability of the reactionet lasso to complement a set of known reactions for the 70 reaction TRAIL induced apoptosis cascade. Specifically, we defined six modules for this cascade following [21], and assumed a limited set of 22 reaction candidates connecting these modules (S8 Fig) and 33 uniformly distributed time points (S1 Dataset). For step 1 of the reactionet lasso all possible unary and binary reactions between components within modules and the module connecting reactions serve as candidate reactions for structure learning, totaling 6828 reactions.

**Fig 6:**
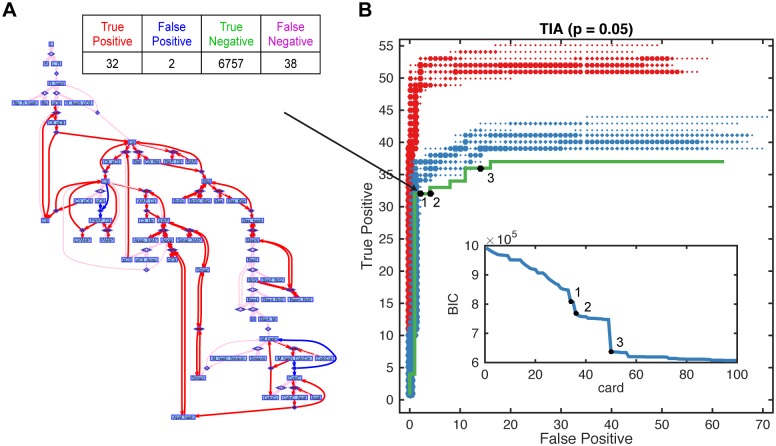
Structure learning with prior knowledge for the 70 component TRAIL induced apoptosis cascade. Structure learning performance for 10^5^ single cell trajectories and 33 time points and capture efficiency *p* = 0.05. Empirical moment gradients estimated with splines. (A) Example of a recovered graph for the setting above and no prior knowledge. True positive reactions are red, false positive reactions are blue, false negative reactions are pink. (B) Regularization paths in terms of true/false positive tradeoff (including prior knowledge reactions, see Results for details) for different prior knowledge situations. Following prior knowledge situations are depicted: no prior knowledge (green). Additional prior knowledge situations comprise ten instances of 10%(blue)/ 50%(red) randomly chosen known reactions. Diameter of dots and color code indicate frequency of solutions with a specific true/false positive tradeoff. Black dots represent solutions coinciding with large improvements of BIC.

In the absence of ground truth it is difficult to identify a regularization strength that achieves a desirable tradeoff between true and false positive reaction discoveries. We evaluated the BIC and report solutions that map to large initial improvements of BIC [22]. Structure learning without prior knowledge on the considered set of reactions achieves 32 true positive at the cost of 2 false positive reactions (10^5^ single cell trajectories, 33 time points, capture efficiency 0.05, Fig 6A). Prior knowledge on a specific reaction was encoded by a positivity constraint on the corresponding reaction rate during all regression steps of the reactionet lasso. We considered different prior knowledge settings: (1) 10% or (2) 50% randomly chosen reactions considered to be known. Settings (1) and (2) were each evaluated using ten different subsets. For 10% known reactions almost 40 (including 7 known) out of 70 reactions are correctly recovered with five or less false positive discoveries (Fig 6B). For 50% known reactions the total number of true positive reactions is beyond 50 (including 35 known). The performance doesn’t depend significantly on the choice of prior reactions. The reactionet lasso enables discovery up to dozens of novel reactions at the cost of few false positive reactions for a large signaling cascade comprising 70 reactions.

While published structure learning approaches are only available for problem instances of sizes hundreds of orders of magnitude smaller, we compared these results to more simple variants of the reactionet lasso procedure, either exhibiting inferior accuracy or exceedingly high computational complexity (S9 Fig).

The above results demonstrate how single cell snapshot time series data and the reactionet lasso can be used to complement prior mechanistic knowledge by a sizable set of candidate reactions that is highly enriched for true positive discoveries, and do so for systems and structure learning tasks of unprecedented size [23, 24].

## Discussion

In this work we introduce the reactionet lasso for comprehensive structure learning of stochastic chemical reaction networks.

Chemical reaction networks constitute a highly detailed and mechanistic description for biological processes and are qualitatively different from other popular network models in biology. These comprise probabilistic graphical models seeking to discover statistical dependencies between measured system components. These approaches range from simple correlation [25] or regression analysis [26], to Bayesian networks [27] or more structured and robust module networks [28] and extensions thereof [29]. In contrast to chemical reaction networks, each of these model classes allows for detection of statistical dependencies without further elucidation of causality relationships and the possibly intricate dependency inducing biochemical mechanism. Physical interaction networks get closer to this goal and complement the information of reaction networks by summarizing measurements of static protein interactions [30].

By virtue of formulating the task of structure learning of chemical reaction networks as a sequence of convex optimization problems, this procedure is able to assess an unprecedented number of potential network topologies without need for explicit enumeration [23, 24]. We demonstrate the utility of the method for ab initio structure learning of whole signaling cascades such as the apoptotic receptor subunit. The reactionet lasso originally integrates a moment based description of stochastic reaction networks with sparse regression approaches via a gradient matching to achieve an efficient and scalable structure learning procedure, overcoming the limitations of available methods for structure learning which either explicitly enumerate a small set of models or greedily search for locally optimal topologies [8, 31]. Recent generic sparse regression approaches for identification of general nonlinear dynamical systems are in principle applicable for structure learning of biological reaction networks [9]. However, these approaches, in contrast to the reactionet lasso, do not take into account their (1) foundation in the Chemical Master Equation, (2) heteroscedastic and parameter dependent noise structure, as well as (3) parameter ranges varying across many scales, therefore failing to achieve competitive structure learning performance (S9 Fig).

The challenging structure learning task crucially depends on sensible experimental design yielding informative data. A central design choice concerns the selection of time points recording the relevant dynamical changes of the process of interest. These are typically chosen from prior knowledge or preliminary dense snapshot time series experiments with a cheap readout, such as population based instead of single cell measurements. Another important experimental parameter concerns the number of single cell snapshots. Our benchmarks advocate for having at least thousands of snapshots per time point. Flow cytometry experiments easily achieve snapshot counts in the order of 10^5^. For single cell transcriptomics experiments it seems advisable to resort to novel droplet based techniques achieving > 10^4^ single cell snapshots per experiment [32, 33].

Structure learning performance of the reactionet lasso depends on the accuracy of the gradient estimates from the time series snapshots. We find that estimates based on gradients obtained by rather simple approaches such as finite difference approximations or spline curve fitting achieve competitive performance. Improvements are conceivable by resorting to other techniques specifically designed for gradient estimation in differential equation systems [34, 35]. These approaches jointly fit parameters of the curve fitting procedure and the differential equation system. The success of this strategy relies on considering a problem instance where the differentiation equation systems strongly constrains the state space. However, the problem instances we consider for systematic structure learning with the reactionet lasso assume a differential equation system defined by the moment equation for all possible unary and binary reactions. Such a system will by definition impose little constraints on the state space. Gradient matching approaches would therefore have to be adapted to avoid expected parameter overfitting resulting from their application to problem instances with such an expressive differential equation system.

For this proof of principle study, we consider single time series experiments. Reactionet lasso analysis easily accommodates multiple replicates or perturbation experiments such as dose responses. Specifically, condition specific response vectors *b*_*k*_ and design matrices *A*_*k*_ for each condition k are utilized to construct a problem instance by concatenation. For this problem instance reactionet lasso can be applied as described (see also S4 Text). Additional experiments are expected to enhance structure learning performance. Indeed, we observe that this is the case for incorporating additional replicate time series experiments (S10 Fig).

The reactionet lasso is able to recover a significant proportion of missing reactions in various settings. However, integration of the moment equations for the component means assuming this set is not always able to recover the observed temporal dynamics of the system. This situation arises for instance when a single pivotal true reaction is missed and therefore precludes the correct reconstruction of downstream component dynamics. We frequently encounter this situation in the ab initio structure learning scenario (S11 Fig). This scenario though constitutes an artificial setting that we only report for a proof of concept of the reactionet lasso. Real world applications comprise prior knowledge about true reactions, typically comprising specifically those pivotal reactions. It turns out that we achieve good reconstruction of integrated trajectories for the structure learning settings assuming prior knowledge (S11 Fig).

Until now we consider reaction systems which obey mass action kinetics. Systems of this kind can be easily translated into a series of moment equations which depend linearly upon the reaction rates However, systems with non-mass action kinetics, such as Michaelis-Menten, can still be addressed with the reactionet lasso. While appropriate moment closure approximations for certain rational rate law kinetics preserve convexity of the reactionet lasso objective [36], generally such kinetics might yield non-convex optimization problems that would have to be dealt with using appropriate optimization techniques.

The reactionet lasso generates a single point estimate for the optimal, sparse reaction network that might neglect other reasonable candidate network structures. Thus, it will be interesting to perform further in-depth analysis of the resulting network structures, for instance with Markov Chain Monte Carlo sampling techniques.

For our study we assume that all relevant molecular components can be measured. Many biological applications, however, do not allow monitoring all relevant components, as for instance antibodies might only be available for a subset of components of a signaling cascade. While the aim of our study was to demonstrate proof of concept for large scale structure learning of chemical reaction networks, it will be possible to account for missing measured components by either augmenting the model by introducing latent variables or ‘lumping’ them into more complex non-mass action reaction mechanisms [37].

The reactionet lasso can be applied in its current form to systems where a significant proportion of relevant components can be measured. Considering the steady advance of single-cell technologies, we expect an increasing number of cellular signaling and metabolic processes to be assayed at single-cell resolution. While mass cytometry approaches allow for measurement of more than 30 protein components, sufficient e.g. to substantially map out the T cell receptor, epidermis growth factor and apoptosis signaling cascades, single-cell RNA sequencing opens the prospect of achieving genome-wide transcriptomic snapshots of single cells. Thus we anticipate a surge of relevant data in the near future for which the reactionet lasso can straightforwardly be applied for systematic and comprehensive structure learning of the underlying reaction networks, with direct implications for systems biology and health by providing quantitative and predictive models for scientific insight and rational intervention design.

## Methods

### Experimental setting: Single cell time series snapshot data

We assume time series data with single cell resolved population snapshots obtained at discrete time points. We denote by *C* the number of cells measured per experiment, *T* the number of time points at which measurements were performed and N the number of components (e.g. proteins) measured in each cell. For each measurement time point *t* = 1, …, *T* for a cell *c* = 1, …, *C* we denote a vector of measured *N* protein abundances $x_{t}^{c}=\left\{x_{t,1}^{c},\ldots ,x_{t,N}^{c}\right\}$. Therefore at each time point *t* vectors $x_{t,1}^{c},\ldots ,x_{t,N}^{c}$ represent a sample from a high-dimensional distribution, which evolves according to the Chemical Master Equation.

### Moment equations for the Chemical Master Equation

We assume a biochemical reaction network of *N* different chemical species with abundances *X*_1_, …, *X*_*N*_ involved in *L* reactions. Each reaction *l* is characterized by stoichiometry vector *s*_*l*_ and propensity function *a*_*l*_(**x**; *k*_*l*_) with **x** representing the collection of species abundances (system state) and *k*_*l*_ the reaction rate. In our work we consider systems described by mass action kinetics, resulting propensities *a*_*l*_(**x**; *k*_*l*_) = *k*_l_*g_*l*_(**x**), where *g*_*l*_(**x**) is a known function of the system’s state. The state of the system evolves probabilistically according to the possible reactions, with probability *P*(**x**, *t*) of occupying state **x** at time *t*. The probabilistic evolution of the system’s state is described by the Chemical Master Equation:

$$\begin{array}{c} \frac{P(x,t)}{dt}=\Sigma_{l=1}^{L}P\left(x-s_{l},t\right)a_{l}\left(x-s_{l}\right)-P\left(x,t\right)a_{l}\left(x\right). \end{array}$$ (8)

We denote by $M_{r}=M_{r_{1},\ldots ,r_{N}}=E{\left(X_{1}-EX_{1}\right)}^{r_{1}}\ldots {\left(X_{N}-EX_{N}\right)}^{r_{N}}$ the central moment of order **r** = (*r*_1_, …, *r*_*N*_). The moment generating function of the probability distribution *P*(**x**, *t*) can be used for the derivation of moment equations [38]. Assuming mass action kinetics, we obtain Eq 1 for the time evolution of a central moment (see also S1 Text).

### Gradient matching for parameter estimation of ordinary differential equation systems

Gradient matching approaches avoid costly integration by instead interpolating the discrete snapshot time series data and estimating the empirical moment gradients *M*_**r**_(*t*), rendering the initial ODE system an algebraic equation system with the parameters as unknowns. This formulation further eliminates the need for moment closure, in contrast to integration based techniques. Previously, gradients have been estimated with spline interpolators [34, 39, 40], Gaussian processes [35, 41] or finite difference approximations [6]. Parameter estimation has been performed by least squares minimization [34, 39, 40] or by approximation of the parameter posterior [6, 35, 41]. While deterministic chemical reaction networks frequently served as application settings for gradient matching schemes, only little attention has been paid to networks with stochastic dynamics [6, 11].

We used and compared cubic spline interpolators (spline) and finite difference approximations (FDS) to estimate empirical moment gradients for the *M*_**r**_(*t*) of the moment equations. As a ground truth estimate for simulated data, we use a smoothed finite difference approximation of the single cell trajectories at the evaluation point of interest (smooth). Gradient estimates are obtained via a smoothing procedure that relies on a sliding window estimate of finite differences on the simulated trajectories using the smoothing function “smooth” in Matlab.

### Moment estimation for noisy single cell data

Single cell data such as obtained from flow/mass cytometry and single cell sequencing exhibit measurement noise. These technologies each detect a random fraction of the total molecular content of every individual cell. This relationship between the measurement signal and cellular analyte abundance has been frequently modeled by a binomial distribution *Bi*(*X*, *p*) whose success probability *p* corresponds to the capture efficiency for the analyte present at amount *X* [42, 43]. We have devised an estimator to subtract the misleading measurement noise component to provide the reactionet lasso with the appropriate noise-correct empirical moment estimates for structure learning.

We assume that measurement noise can be represented by the following binomial model. Let *X* represent the true abundance of one species at a given time point. Let *X*^*obs*^ be the corresponding measured signal, such that *X*^*obs*^ ∼ *Bi*(*X*, *p*), where *p* is the capture efficiency. The binomial noise model allows for specifying the following analytical relationships between the first and second order moments of *X* and *X*^*obs*^:

$$\begin{array}{c} E\left[X^{obs}\right]=pE\left[X\right], \end{array}$$ (9)

$$\begin{array}{c} E\left[{\left(X^{obs}\right)}^{2}\right]=p\left(1-p\right)E\left[X\right]+p^{2}E\left[X^{2}\right], \end{array}$$ (10)

$$\begin{array}{c} Var\left[X^{obs}\right]=p\left(1-p\right)E\left[X\right]+p^{2}Var\left[X\right], \end{array}$$ (11)

$$\begin{array}{c} \text{cov}\left(X_{1}^{obs},X_{2}^{obs}\right)=p^{2}\text{cov}\left(X_{1},X_{2}\right), \end{array}$$ (12)

For a derivation see S3 Text. We assume that the capture efficiency *p* of the single cell instrument is known [42, 43] and estimates the empirical moments of *X* on the basis of the empirical moments of *X*^*obs*^ by solving the above equations for the respective moment of *X*. The resulting moment estimates are then used in the regression procedure described above to perform structure learning.

## Supporting Information

S1 Fig Structure learning performance of the *reactionet lasso*. 10^5^ single cell trajectories evaluated at 13 time points for (A) apoptotic receptor subunit (no measurement noise); (B) the enzymatic system. Empirical moment gradients estimated with cubic splines. Solution selected with Bayesian Information Criteria (BIC).
(TIF)

S2 Fig Regularization paths in terms of true/false positive tradeoff over different data availability situations. Results for *reactionet lasso* application to apoptotic receptor subunit (no measurement noise). (A-B) Empirical moment gradients estimated with “smooth” procedure: (A) 10^5^ single cell trajectories evaluated at different amount of time points (tp) as indicated in the legend. (B) Different number of single cell trajectories: 10^3^, 10^4^, 10^5^ evaluated at thirteen time points. (C-E) Results for different empirical moment gradient estimates: smooth (red), splines (blue), FDS (green) for different amount of time points: 28 (C), 13 (D), 7 (E).
(TIF)

S3 Fig Regularization paths in terms of true/false positive tradeoff over different data availability situations. Results for *reactionet lasso* application to enzymatic system (no measurement noise). (A-B) Empirical moment gradients estimated with “smooth” procedure: (A) 10^5^ single cell trajectories evaluated at different amount of time points (tp) as indicated in the legend. (B) Different number of single cell trajectories: 10^3^, 10^4^, 10^5^ evaluated at thirteen time points. (C-E) Results for different empirical moment gradient estimates: smooth (red), splines (blue), FDS (green) for different amount of time points: 28 (C), 13 (D), 7 (E).
(TIF)

S4 Fig Overlay of five regularization paths in terms of true/false positive tradeoff over different data availability situations. Results for *reactionet lasso* application to apoptotic receptor subunit (p = 0.05) with 10^5^ trajectories. Results for different empirical moment gradient estimates: splines (red), FDS (blue) for different amount of time points: 28 (A), 13 (B), 7 (C).
(TIF)

S5 Fig Structure learning performance of the *reactionet lasso*. 10^5^ single cell trajectories evaluated at 13 time points for apoptotic receptor subunit (*p* = 0.05). Empirical moment gradients estimated with cubic splines. Solution selected with Bayesian Information Criteria (BIC).
(TIF)

S6 Fig Analysis of standard deviation of moment and stoichiometric moment function estimates for high order moments for different sample sizes. Results for application to apoptotic receptor subunit (p = 0.05). (A) Absolute values of standard deviation of moment estimate estimated from bootstrap for the apoptotic receptor subunit with no noise with 10^5^ (red), 10^4^ (blue), 10^3^ (green) trajectories, 13 time points. (B) Relative change of standard deviation of the moment estimates with decreasing number of trajectories compared to 10^5^. (C) Corresponding absolute and relative change of standard deviation of design matrix estimate (with stoichiometric moment functions as entries) with decreasing number of samples compared to 10^5^.
(TIF)

S7 Fig Overlay of five regularization paths in terms of true/false positive tradeoff over different data availability situations. Results for *reactionet lasso* application to apoptotic receptor subunit for uniform selection of time points. Results for different empirical moment gradient estimates: splines (red), FDS (blue) for different amount of time points and different levels of noise: 28 (A, D), 13 (B, E), 7 (C, F).
(TIF)

S8 Fig Original reaction network of TRAIL induced apoptosis. Different modules colored in different colors. Reactions connecting the models depicted in gray.
(TIF)

S9 Fig Comparison of the *reactionet lasso* with various baseline procedures. RL = *reactionet lasso*; STlsq = sequential thresholded regression, TF = Topological filtering. All methods applied to Moment Equations of 1st and 2nd order correspondingly. Results for: **(A)** the apoptotic receptor subunit with noise (p = 0.05) with 10^5^ trajectories, 13 time points; **(B)** TRAIL-induced apoptosis with noise (p = 0.05) with 10^5^ trajectories, 33 time points. TF2 was interrupted after 2h hours and didn’t produce any solution in the range of cardinality represented on the plot.
(TIF)

S10 Fig Results for application of the matrix concatenation strategy for *reactionet lasso* for the case of multiple replicates. 5 replicates of the apoptotic receptor subunit (*p* = 0.05) were generated with 10^5^ single cell trajectories each evaluated at 13 time points. Red dots correspond to different replicates. Size of the dot proportional to the frequency of the solution between the replicates. Blue line corresponds to the strategy of concatenating design and response matrices.
(TIF)

S11 Fig Recovery of the dynamics of mean trajectories by the *reactionet lasso*. Red: observed data for 10^5^ single cell trajectories evaluated at 13 time points for apoptotic receptor subunit without measurement noise. Solution selected with AIC for two distinct scenarios: *ab initio* learning (blue), *a priori* specified reaction identified false negative in *ab initio* learning setting (green).
(TIF)

S1 Text Moment expansion. (PDF)

S2 Text Information criteria. (PDF)

S3 Text Inference of binomial noise correction for empirical moments. (PDF)

S4 Text Biological replicates. (PDF)

S1 Dataset Time points selection for TRAIL induced apoptosis signaling cascade. (PDF)
