# Supplementary figures and images for: Sparse Regression Based Structure Learning of Stochastic Reaction Networks from Single Cell Snapshot Time Series
Source: PLoS Comput Biol. 2016 Dec 6;12(12):e1005234. doi: 10.1371/journal.pcbi.1005234 (PMC5140059; doi:10.1371/journal.pcbi.1005234)

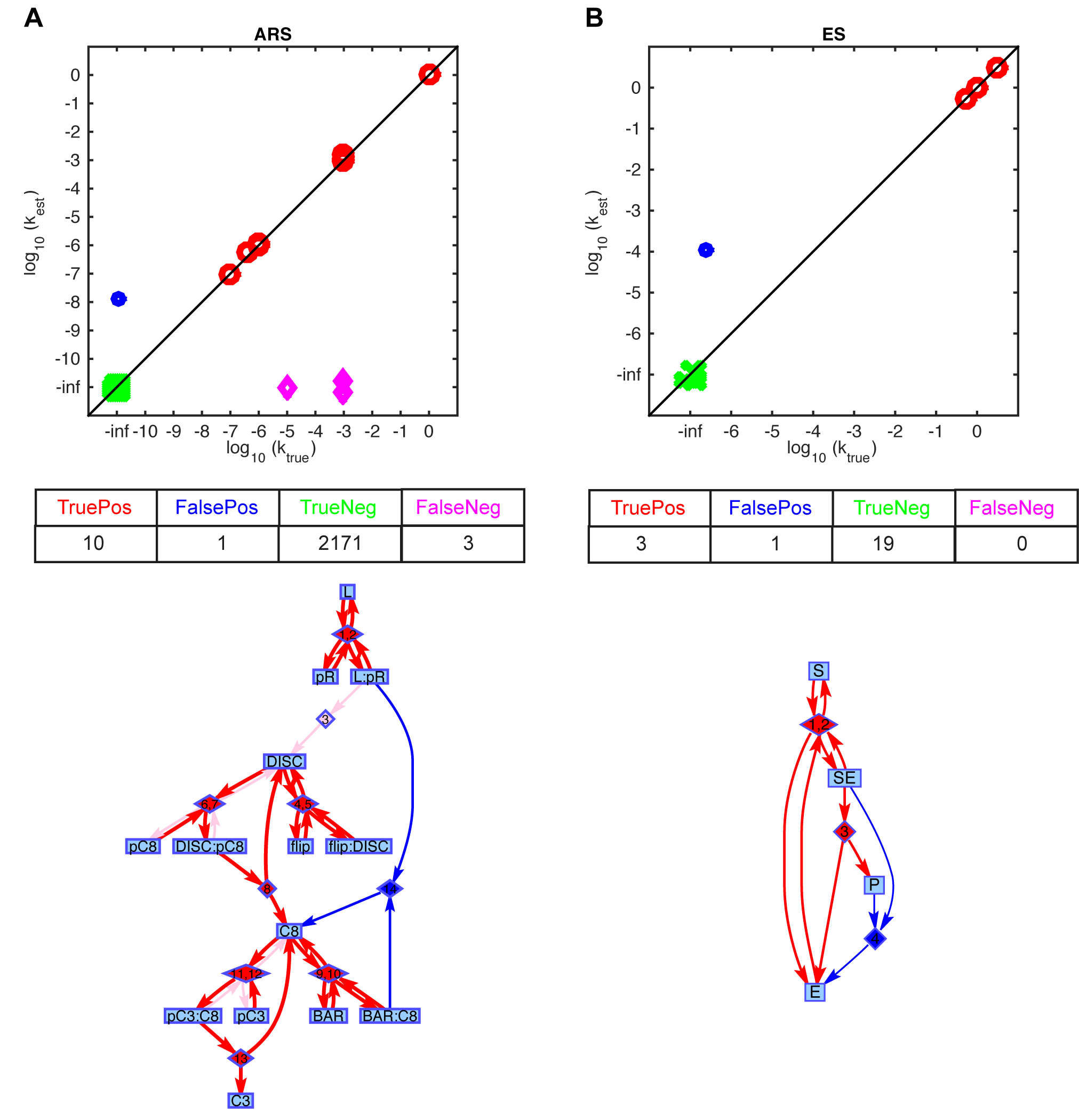

Supplement: S1 Fig — 105 single cell trajectories evaluated at 13 time points for (A) apoptotic receptor subunit (no measurement noise); (B) the enzymatic system. Empirical moment gradients estimated with cubic splines. Solution selected with Bayesian Information Criteria (BIC). (TIF) [file pcbi.1005234.s001.tif]

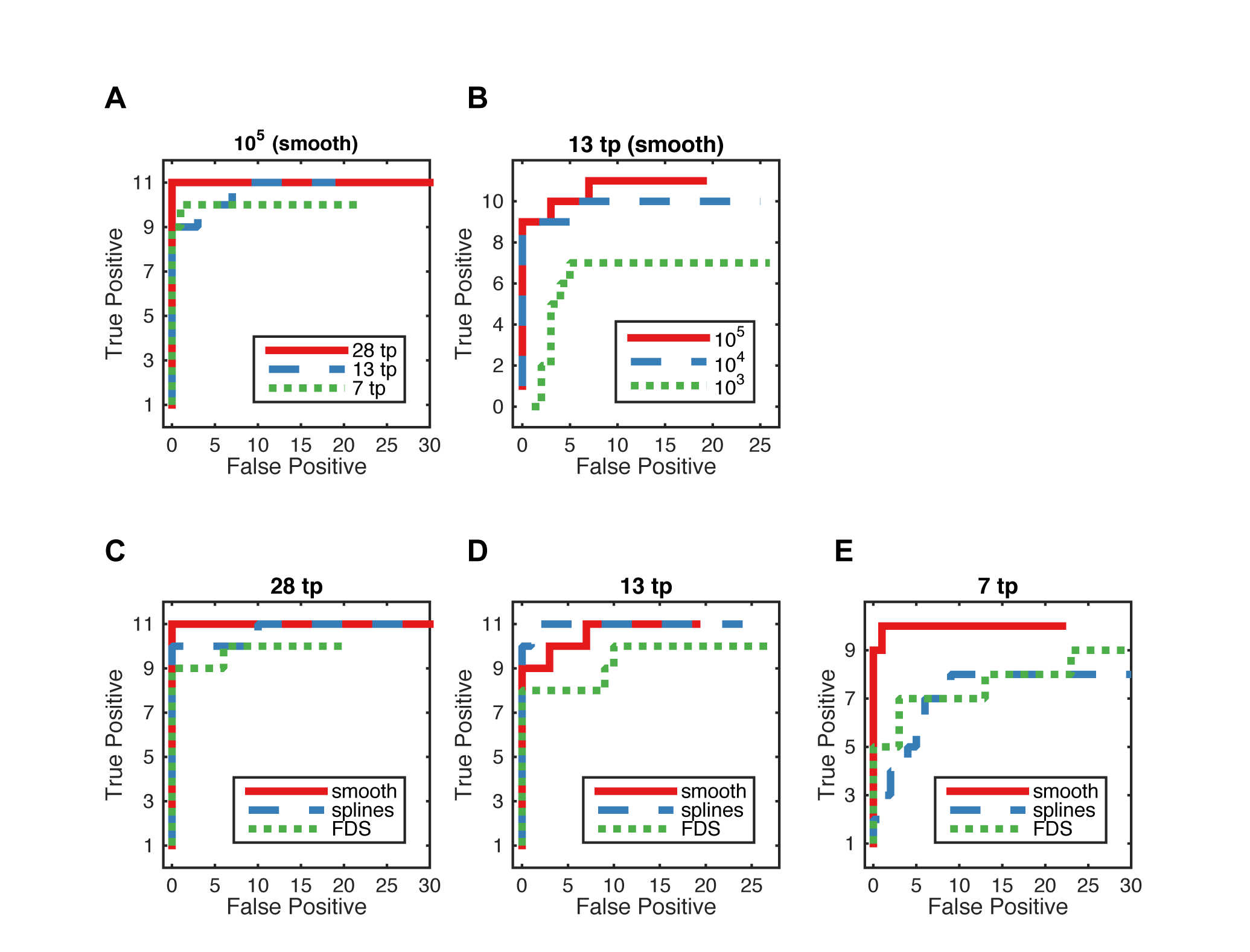

Supplement: S2 Fig — Results for reactionet lasso application to apoptotic receptor subunit (no measurement noise). (A-B) Empirical moment gradients estimated with “smooth” procedure: (A) 105 single cell trajectories evaluated at different amount of time points (tp) as indicated in the legend. (B) Different number of single cell trajectories: 103, 104, 105 evaluated at thirteen time points. (C-E) Results for different empirical moment gradient estimates: smooth (red), splines (blue), FDS (green) for different amount of time points: 28 (C), 13 (D), 7 (E). (TIF) [file pcbi.1005234.s002.tif]

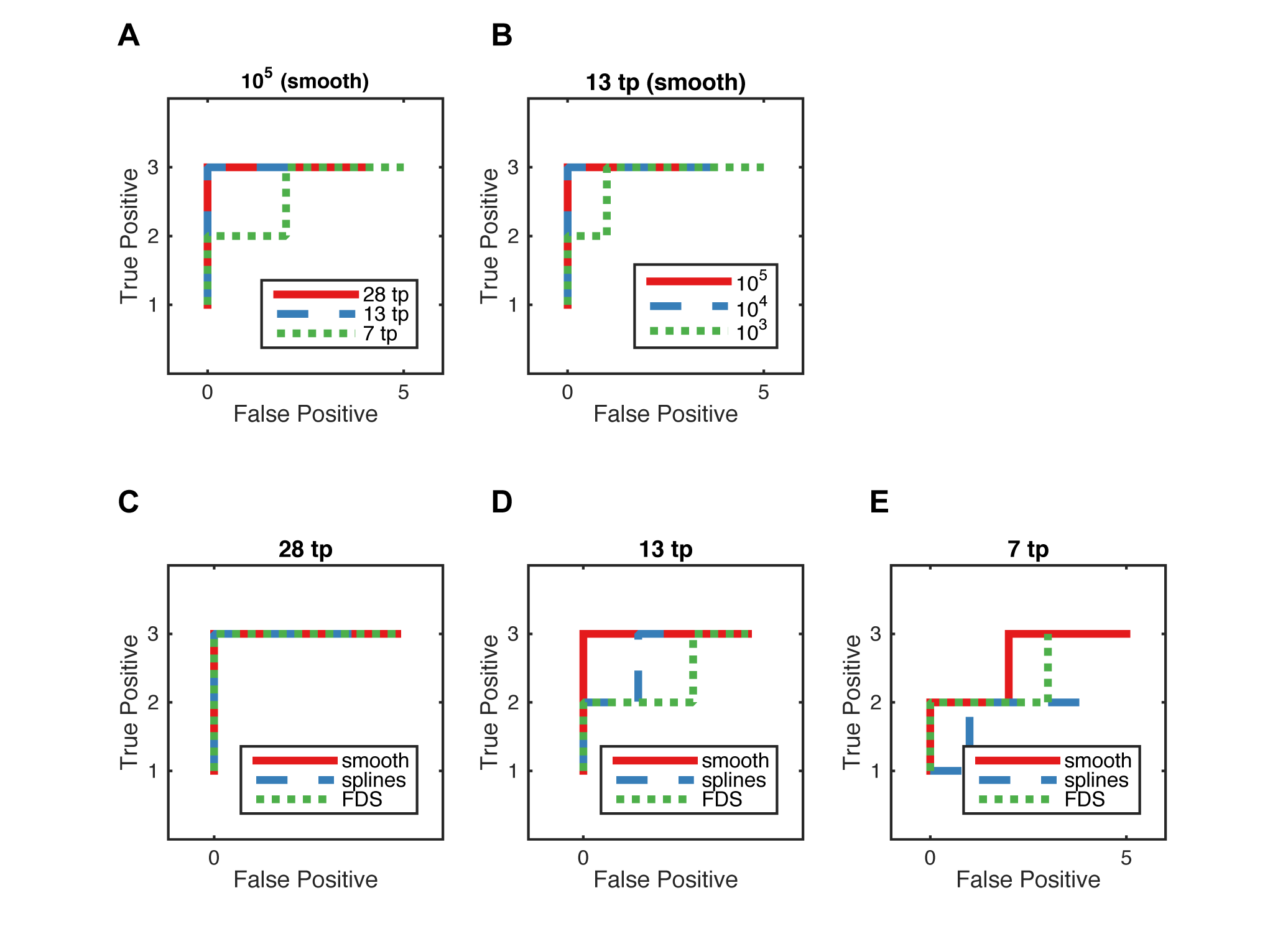

Supplement: S3 Fig — Results for reactionet lasso application to enzymatic system (no measurement noise). (A-B) Empirical moment gradients estimated with “smooth” procedure: (A) 105 single cell trajectories evaluated at different amount of time points (tp) as indicated in the legend. (B) Different number of single cell trajectories: 103, 104, 105 evaluated at thirteen time points. (C-E) Results for different empirical moment gradient estimates: smooth (red), splines (blue), FDS (green) for different amount of time points: 28 (C), 13 (D), 7 (E). (TIF) [file pcbi.1005234.s003.tif]

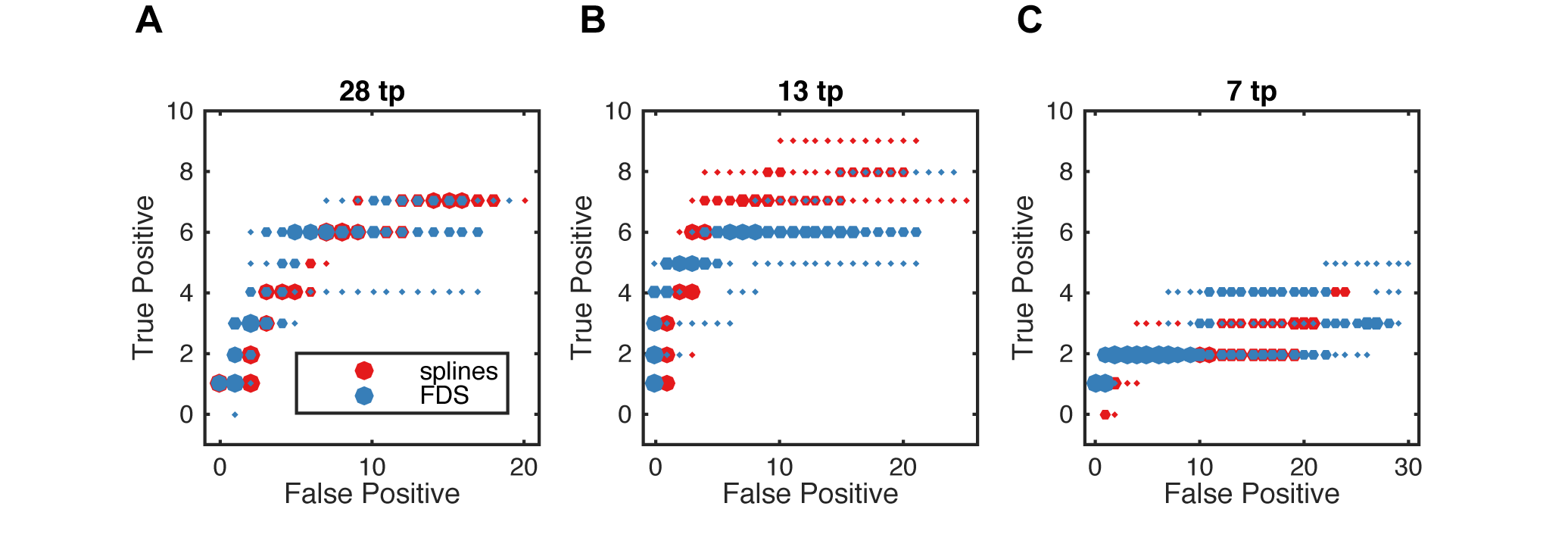

Supplement: S4 Fig — Results for reactionet lasso application to apoptotic receptor subunit (p = 0.05) with 105 trajectories. Results for different empirical moment gradient estimates: splines (red), FDS (blue) for different amount of time points: 28 (A), 13 (B), 7 (C). (TIF) [file pcbi.1005234.s004.tif]

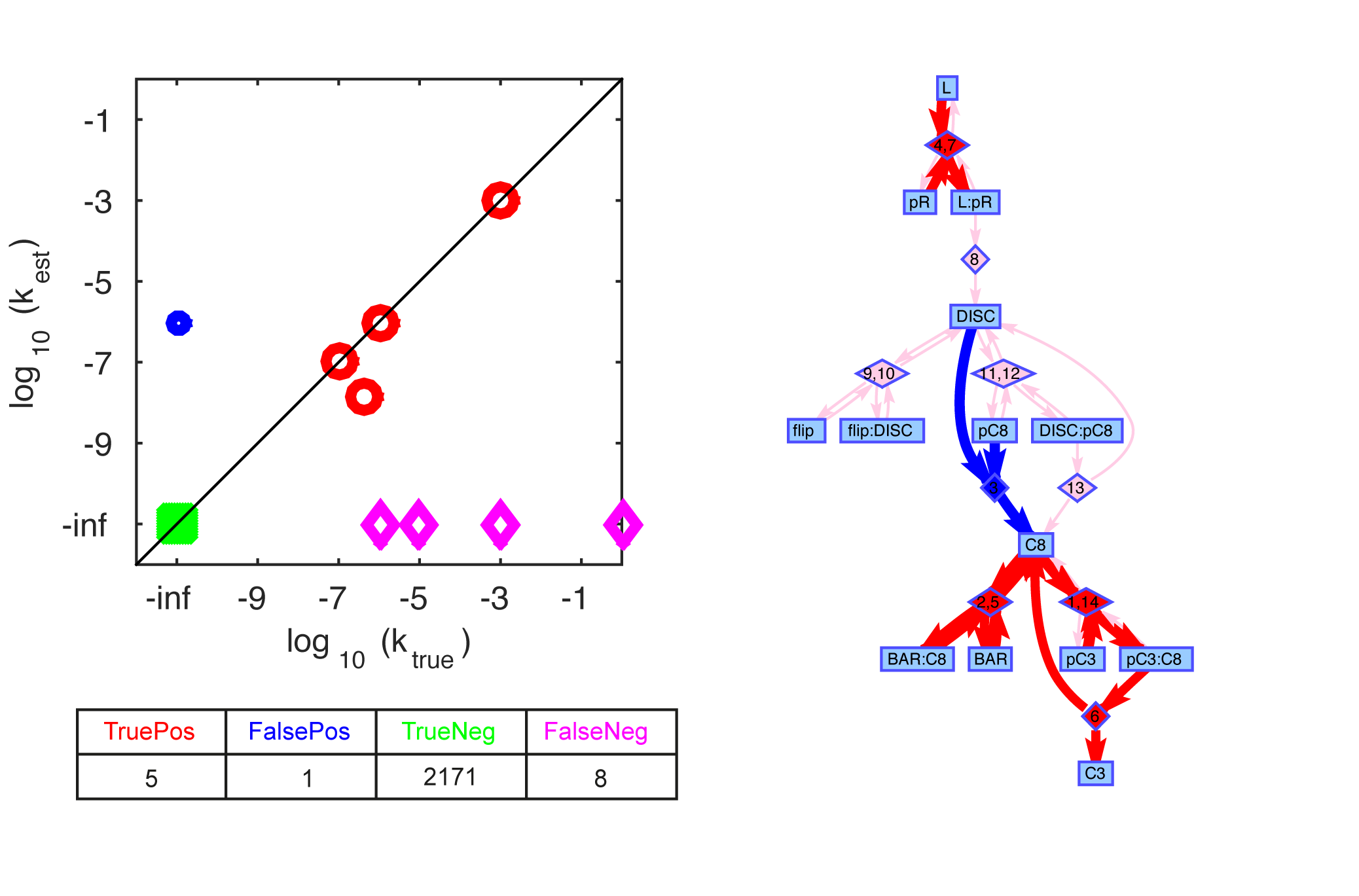

Supplement: S5 Fig — 105 single cell trajectories evaluated at 13 time points for apoptotic receptor subunit (p = 0.05). Empirical moment gradients estimated with cubic splines. Solution selected with Bayesian Information Criteria (BIC). (TIF) [file pcbi.1005234.s005.tif]

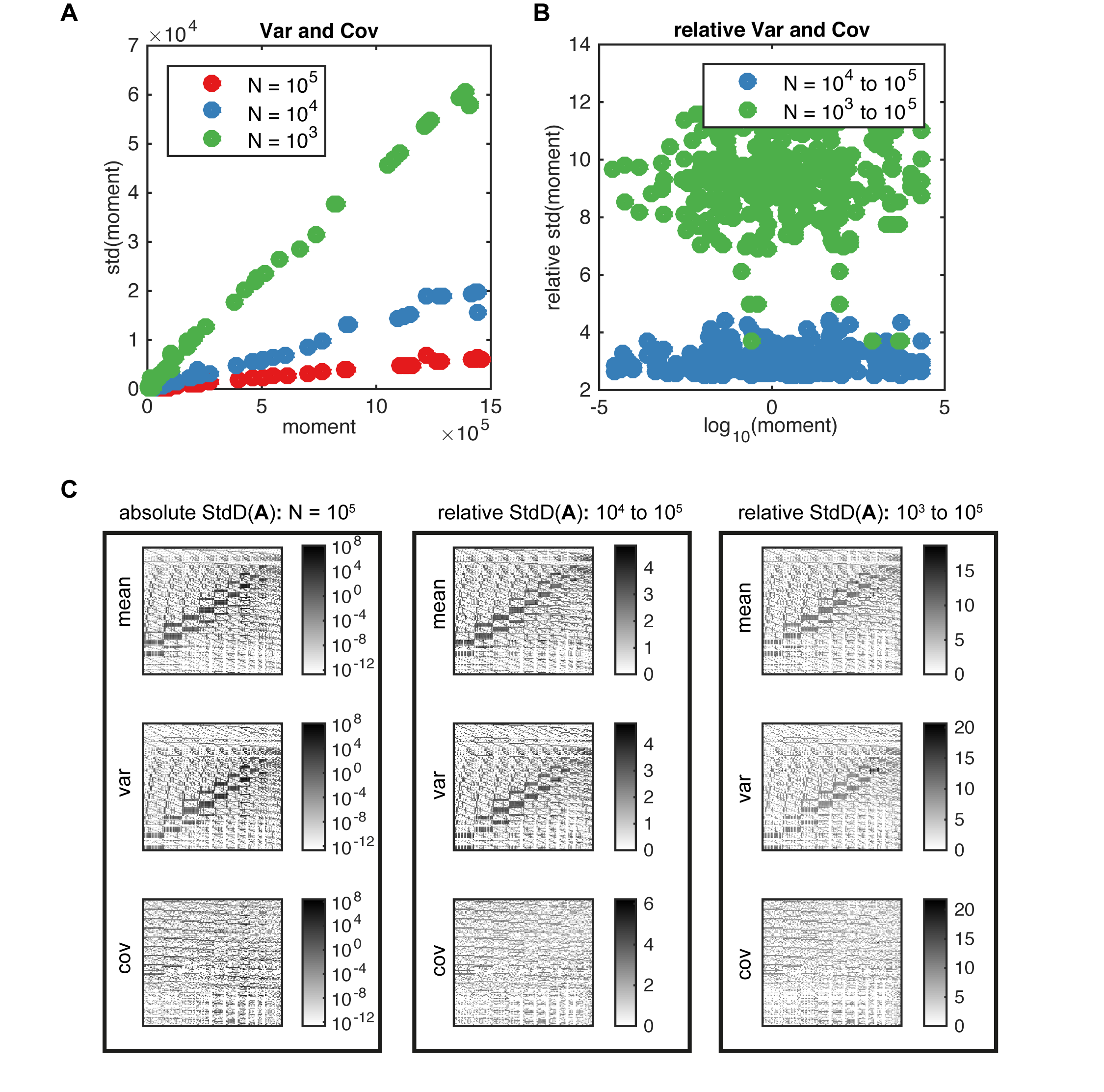

Supplement: S6 Fig — Results for application to apoptotic receptor subunit (p = 0.05). (A) Absolute values of standard deviation of moment estimate estimated from bootstrap for the apoptotic receptor subunit with no noise with 105 (red), 104 (blue), 103 (green) trajectories, 13 time points. (B) Relative change of standard deviation of the moment estimates with decreasing number of trajectories compared to 105. (C) Corresponding absolute and relative change of standard deviation of design matrix estimate (with stoichiometric moment functions as entries) with decreasing number of samples compared to 105. (TIF) [file pcbi.1005234.s006.tif]

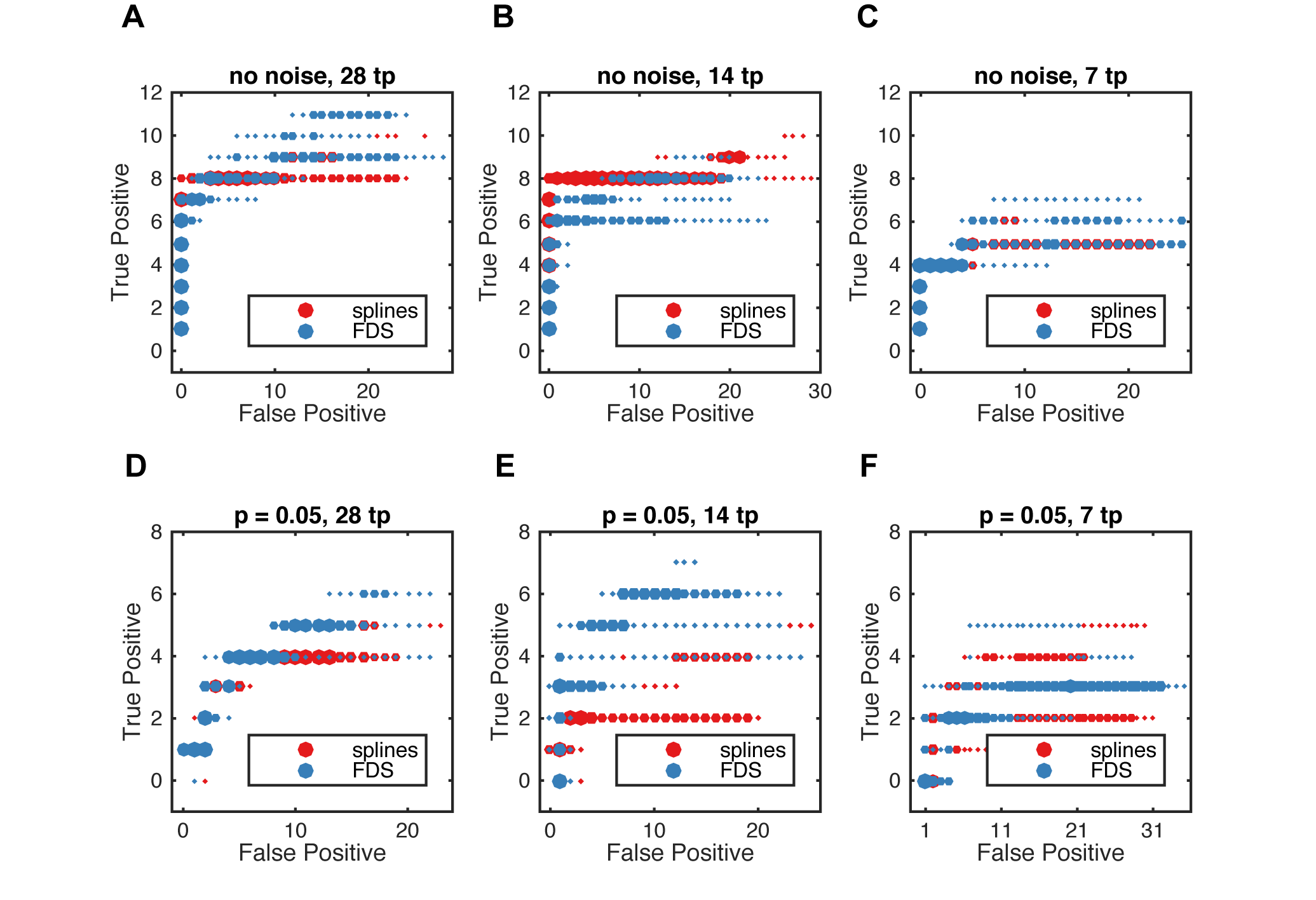

Supplement: S7 Fig — Results for reactionet lasso application to apoptotic receptor subunit for uniform selection of time points. Results for different empirical moment gradient estimates: splines (red), FDS (blue) for different amount of time points and different levels of noise: 28 (A, D), 13 (B, E), 7 (C, F). (TIF) [file pcbi.1005234.s007.tif]

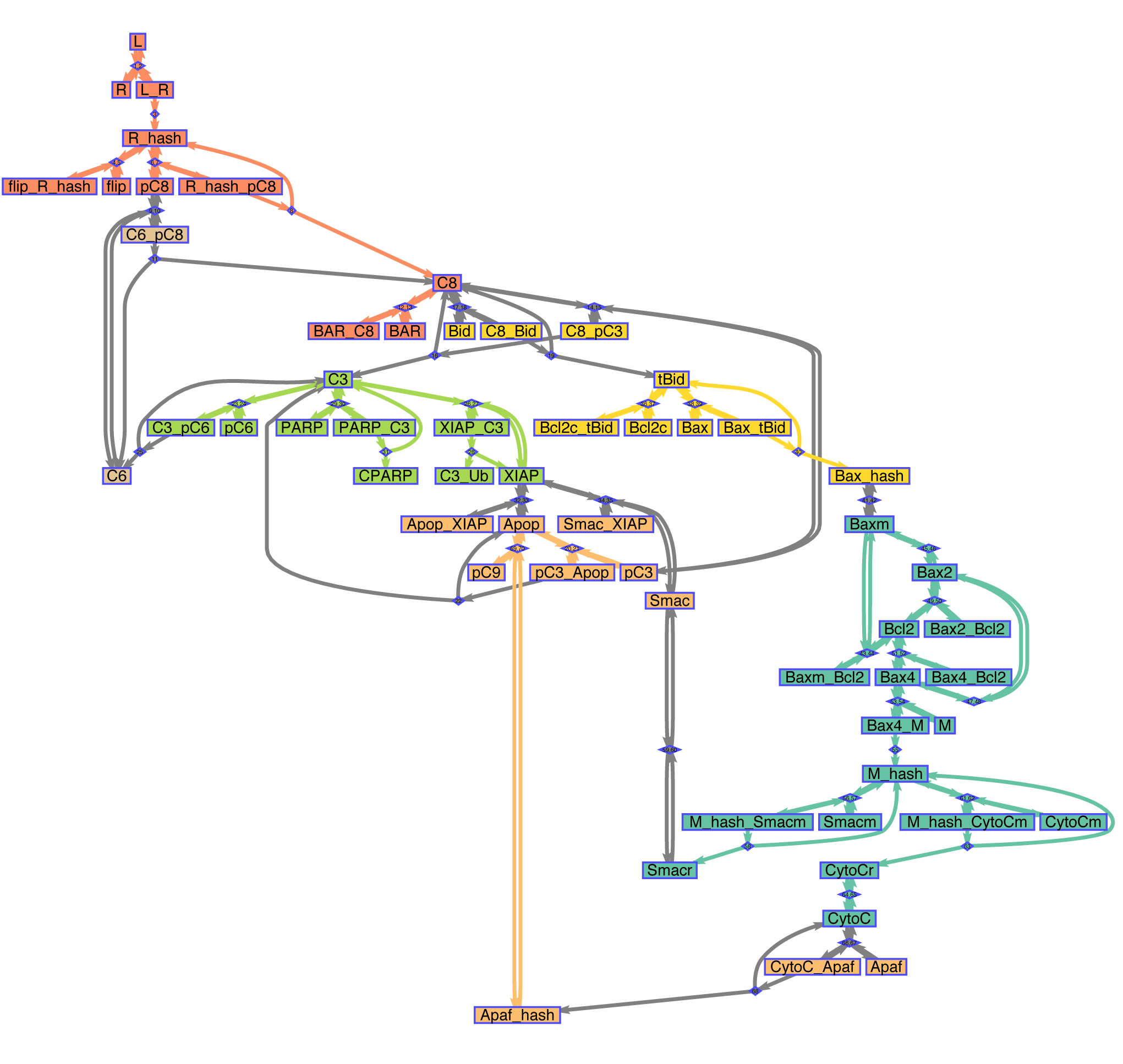

Supplement: S8 Fig — Different modules colored in different colors. Reactions connecting the models depicted in gray. (TIF) [file pcbi.1005234.s008.tif]

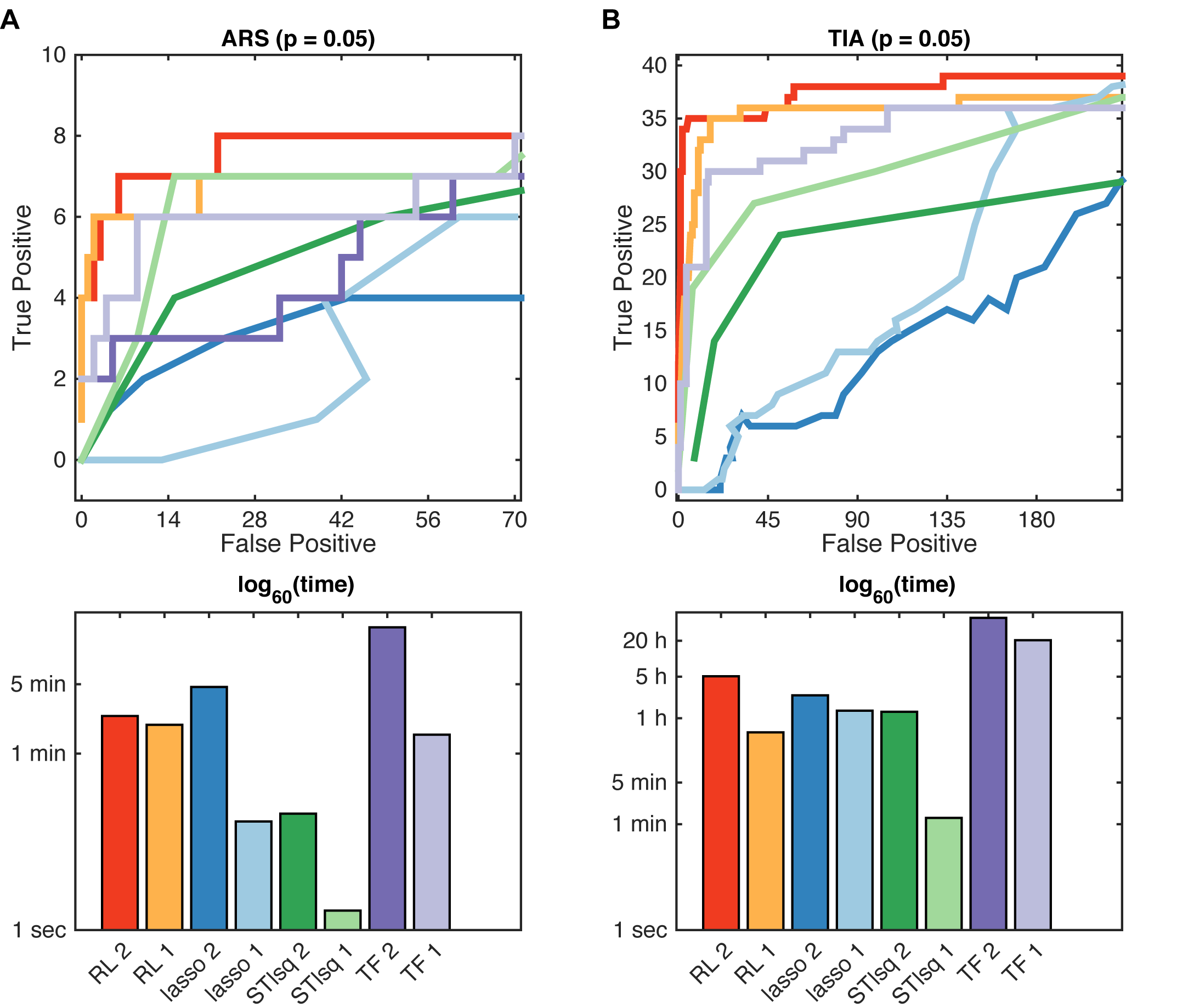

Supplement: S9 Fig — RL = reactionet lasso; STlsq = sequential thresholded regression, TF = Topological filtering. All methods applied to Moment Equations of 1st and 2nd order correspondingly. Results for: (A) the apoptotic receptor subunit with noise (p = 0.05) with 105 trajectories, 13 time points; (B) TRAIL-induced apoptosis with noise (p = 0.05) with 105 trajectories, 33 time points. TF2 was interrupted after 2h hours and didn’t produce any solution in the range of cardinality represented on the plot. (TIF) [file pcbi.1005234.s009.tif]

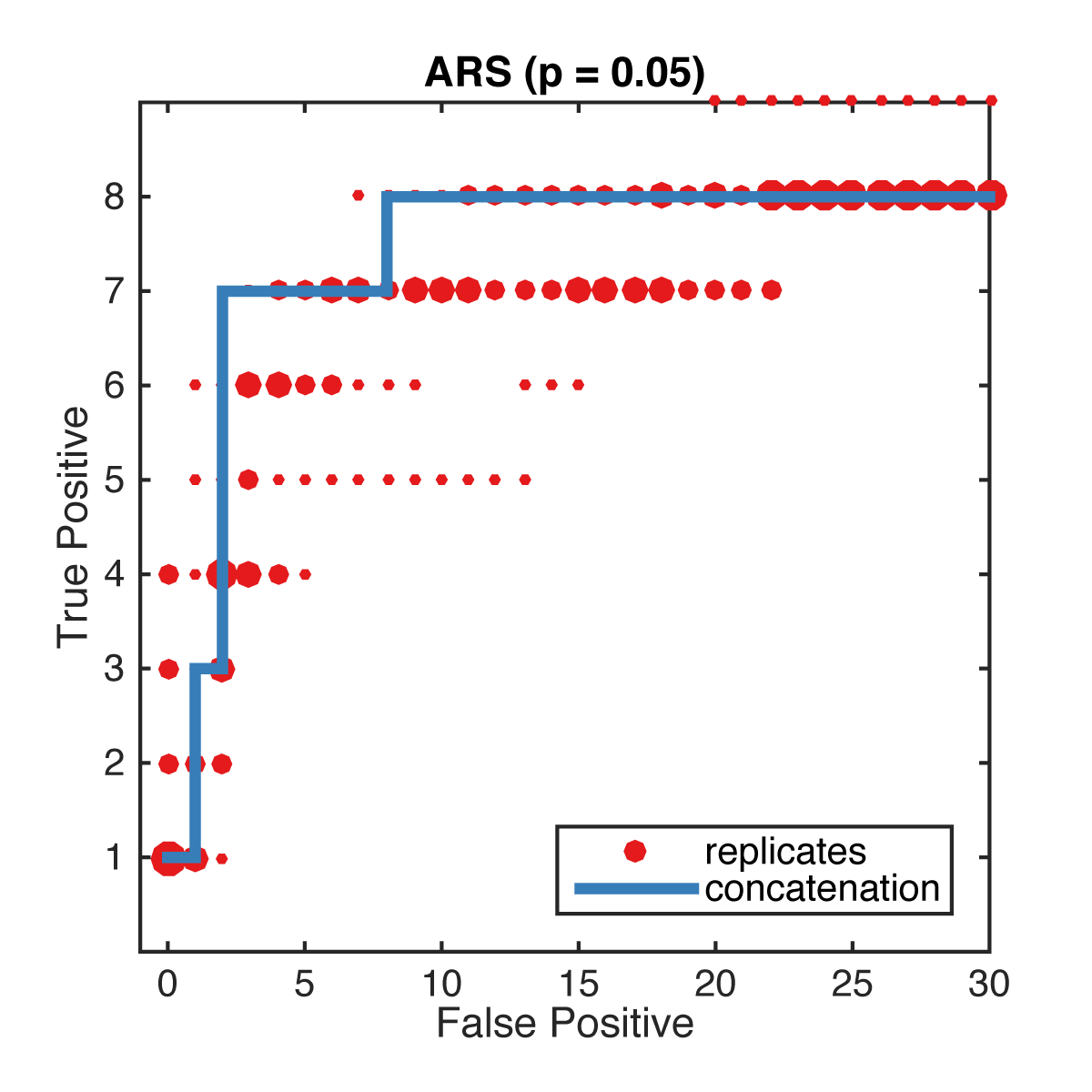

Supplement: S10 Fig — 5 replicates of the apoptotic receptor subunit (p = 0.05) were generated with 105 single cell trajectories each evaluated at 13 time points. Red dots correspond to different replicates. Size of the dot proportional to the frequency of the solution between the replicates. Blue line corresponds to the strategy of concatenating design and response matrices. (TIF) [file pcbi.1005234.s010.tif]

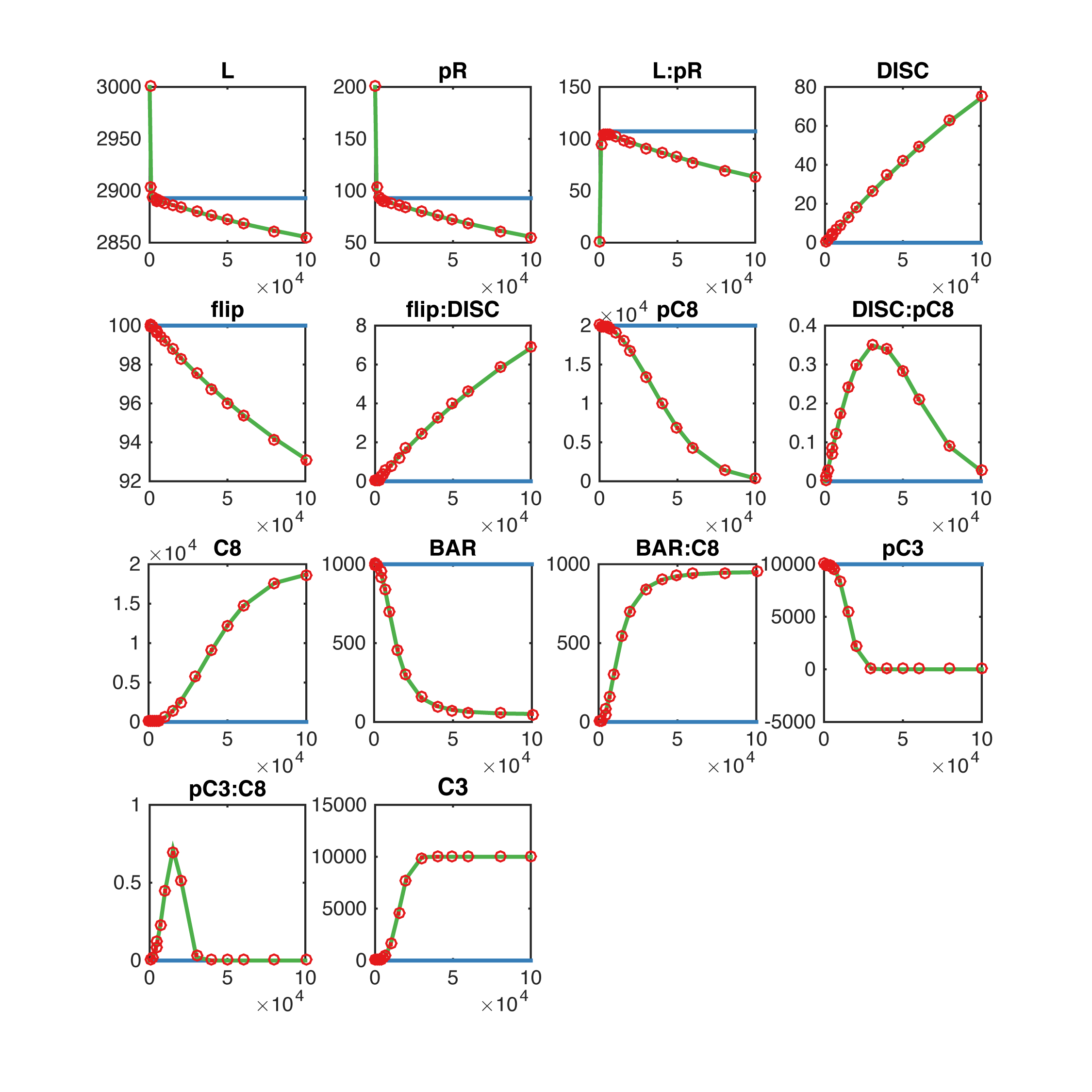

Supplement: S11 Fig — Red: observed data for 105 single cell trajectories evaluated at 13 time points for apoptotic receptor subunit without measurement noise. Solution selected with AIC for two distinct scenarios: ab initio learning (blue), a priori specified reaction identified false negative in ab initio learning setting (green). (TIF) [file pcbi.1005234.s011.tif]
