# Supplementary material for: Sparse Regression Based Structure Learning of Stochastic Reaction Networks from Single Cell Snapshot Time Series
Source: PLoS Comput Biol. 2016 Dec 6;12(12):e1005234. doi: 10.1371/journal.pcbi.1005234 (PMC5140059; doi:10.1371/journal.pcbi.1005234)
Supplement: S1 Text — (PDF) [file pcbi.1005234.s012.pdf]

# S1 Text

**Moment expansion**. Lets consider Stochastic Chemical Reaction Network described in *Methods*. Lets denote stoichiometric matrix of this system as $S = \{s\}_{ij}$. In mass action kinetics propensity function $a_l(\mathbf{x}; k_l)$ can be represented as $a_l(\mathbf{x}; k_l) = k_l * g_l(\mathbf{x})$, where

$$g_l(\mathbf{X}(t)) = \begin{cases} X_a(t)X_b(t), & \text{for bimolecular reactions } R_l : a + b \rightarrow \dots \\ X_a(t), & \text{for unimolecular reactions } R_l : a \rightarrow \dots \end{cases}$$

Following [38] we can derive equations for moment expansion for mass action kinetics for moments of any order. However, for the reactionet lasso we use only moments up to second order, so we provide closed form equations only for these moments. Let denote $\mu_i(t) = \mathbb{E}[X_i(t)]$, $\sigma_i(t) = \mathrm{Var}[X_i(t)]$, $c_{ij}(t) = \mathrm{Cov}(X_i(t), X_j(t))$.

Then dynamics of means follows:

$$\frac{d\mu_i}{dt}(t) = \sum_l k_l * F_{il}(t), \tag{1}$$

where

$$F_{il}(t) = s_{il} * \mathbb{E}[g_l(\mathbf{X}(t))].$$

The dynamics of variances follows:

$$\frac{d\sigma_i}{dt}(t) = \sum_l k_l * F_{(N+i)l}(t), \tag{2}$$

where

$$F_{(N+i)l}(t) = s_{il}^2 * \mathbb{E}[g_l(\mathbf{X}(t))] + 2s_{il} * \mathrm{Cov}(X_i(t), g_l(\mathbf{X}(t))).$$

And the dynamics of covariances follows:

$$\frac{dc_{ij}}{dt}(t) = \sum_l k_l * F_{(N(i+1)+j)l}(t), \tag{3}$$

where

$$F_{(N(i+1)+j)l}(t) = s_{il}^2 * \mathbb{E}[g_l(\mathbf{X}(t))] + s_{il} * \mathrm{Cov}(X_j(t), g_l(\mathbf{X}(t))) + s_{jl} * \mathrm{Cov}(X_i(t), g_l(\mathbf{X}(t))).$$
