# Supplementary material for: Sparse Regression Based Structure Learning of Stochastic Reaction Networks from Single Cell Snapshot Time Series
Source: PLoS Comput Biol. 2016 Dec 6;12(12):e1005234. doi: 10.1371/journal.pcbi.1005234 (PMC5140059; doi:10.1371/journal.pcbi.1005234)
Supplement: S2 Text — (PDF) [file pcbi.1005234.s013.pdf]

S2 Text

Information criteria. The output of the reactionet lasso is a set of nested models. Selection of the optimal solution in terms of true/false positive tradeoff without ground truth still remains an open question. In the current paper we applied different strategies based on information criteria. To asses trade-off of goodness-of-fit and cardinality of the solution we used Akaike information criteria (AIC) or Bayesian information criteria (BIC).

A common selection strategy is to use the minimum of either of these functions. However, we found this strategy suboptimal for reactionet lasso. Evaluation of information criteria turns out to be monotonously improving for many problems, as it was the case for our problem instances. We therefore recommend to use 1st, 2nd etc. maximum of absolute change in the information criteria. Fig.6 demonstrates how absolute change of the BIC corresponds to desirable true/false positive trade-off (black dots labeled "1", "2", "3"). We applied described approach to the regression problem formulation from Eq.3 (see *Results*).
