# Supplementary material for: Sparse Regression Based Structure Learning of Stochastic Reaction Networks from Single Cell Snapshot Time Series
Source: PLoS Comput Biol. 2016 Dec 6;12(12):e1005234. doi: 10.1371/journal.pcbi.1005234 (PMC5140059; doi:10.1371/journal.pcbi.1005234)
Supplement: S3 Text — (PDF) [file pcbi.1005234.s014.pdf]

S3 Text

Inference of binomial noise correction for empirical moments. Lets X a random variable denoting theoretical abundance of a given specie s at a give time point t. Lets assume that as an outcome of an experiment we observe X_{obs}, which follows binomial distribution $Bi(X, p)$ with some fixed probability of success p. This allows us to explicitly formalize connections between moments of X and X_{obs}:

$$\mathbb{E}[X_{\text{obs}}] = \mathbb{E}\,\mathbb{E}[X_{\text{obs}}|X] = \mathbb{E}[Xp] = p\,\mathbb{E}[X] \tag{4}$$

$$\mathbb{E}[X_{\text{obs}}^2] = \mathbb{E}\,\mathbb{E}[X_{\text{obs}}^2|X] = \mathbb{E}[Xp(1-p) + X^2p^2] = p(1-p)\,\mathbb{E}[X] + p^2\,\mathbb{E}[X^2] \tag{5}$$

$$\mathbb{E}[X_{\text{obs}}^2] = \mathbb{E}\,\mathbb{E}[X_{\text{obs}}^2|X] = \mathbb{E}[Xp(1-p) + X^2p^2] = p(1-p)\,\mathbb{E}[X] + p^2\,\mathbb{E}[X^2] \tag{6}$$

$$\text{Var}[X_{\text{obs}}] = \mathbb{E}[X_{\text{obs}}^2] - [\mathbb{E}\,X_{\text{obs}}]^2 = \; = p(1-p)\,\mathbb{E}[X] + p^2\,\mathbb{E}[X^2] - p^2(\mathbb{E}\,X)^2 = p(1-p)\,\mathbb{E}\,X + p^2\text{Var}[X] \tag{7}$$

$$\mathbb{E}[X_{\text{obs, }1}X_{\text{obs, }2}] = \mathbb{E}\,\mathbb{E}[X_{\text{obs, }1}X_{\text{obs, }2}|X_1, X_2] = \mathbb{E}[pX_1pX_2] = p^2\,\mathbb{E}[X_1X_2] \tag{8}$$

$$\begin{aligned}\text{Cov}(X_{\text{obs, }1}, X_{\text{obs, }2}) &= \mathbb{E}[X_{\text{obs, }1}X_{\text{obs, }2}] - \mathbb{E}\,X_{\text{obs, }1}\,\mathbb{E}\,X_{\text{obs, }2} = \\ &= p^2\,\mathbb{E}[X_1X_2] - p\,\mathbb{E}[X_1]p\,\mathbb{E}[X_2] = p^2\text{Cov}(X_1, X_2)\end{aligned} \tag{9}$$
