# Supplementary material for: Sparse Regression Based Structure Learning of Stochastic Reaction Networks from Single Cell Snapshot Time Series
Source: PLoS Comput Biol. 2016 Dec 6;12(12):e1005234. doi: 10.1371/journal.pcbi.1005234 (PMC5140059; doi:10.1371/journal.pcbi.1005234)
Supplement: S4 Text — (PDF) [file pcbi.1005234.s015.pdf]

S4 Text

Biological replicates. As a default, we consider single time series experiments. Reactionet lasso analysis easily accommodates multiple replicates. Specifically, replicate specific response vectors b_k and design matrices A_k for each condition k are utilized to construct a problem instance by concatenation and apply reactionet lasso as described:

Matrix concatenation strategy:

1. Perform bootstrapping of stoichiometric moment function evaluations separately for each of the replicates k.

2. Construct joint regression problem by concatenation:
   response vector $b := [b_1 b_2 \dots b_n]$
   design matrix $A := [A_1 A_2 \dots A_n]$..

3. Run reactionet lasso on the joint regression problem defined by b and A.
