# Supplementary material for: Sparse Regression Based Structure Learning of Stochastic Reaction Networks from Single Cell Snapshot Time Series
Source: PLoS Comput Biol. 2016 Dec 6;12(12):e1005234. doi: 10.1371/journal.pcbi.1005234 (PMC5140059; doi:10.1371/journal.pcbi.1005234)
Supplement: S1 Dataset — (PDF) [file pcbi.1005234.s016.pdf]

S1 Dataset

For TRAIL induced apoptosis signaling cascade inference we used 33 equally distributed time points (in seconds) between 0 and 8 hours: [0, 900, 1800, 2700, 3600, 4500, 5400, 6300, 7200, 8100, 9000, 9900, 10800, 11700, 12600, 13500, 14400, 15300, 16200, 17100, 18000, 18900, 19800, 20700, 21600, 22500, 23400, 24300, 25200, 26100, 27000, 27900, 28800].
